# School environment, sedentary behavior and physical activity in preschool children

**DOI:** 10.1016/j.rppede.2016.02.003

**Published:** 2016

**Authors:** Sara Crosatti Barbosa, Diogo Henrique Constantino Coledam, Antonio Stabelini, Rui Gonçalves Marques Elias, Arli Ramos de Oliveira

**Affiliations:** a Centro de Ciências da Saúde, Universidade Estadual do Norte do Paraná, Jacarezinho, PR, Brazil; b Instituto Federal de São Paulo, Campus Boituva, Boituva, SP, Brazil; c Departamento de Ciências do Esporte, Centro de Educação Física e Esportes, Universidade Estadual de Londrina, Londrina, PR, Brazil

**Keywords:** Environment, Motor activity, Day care, Child, Infrastructure

## Abstract

**Objective:**

To analyze physical activity and sedentary behavior in preschool children during their stay at school and the associated factors.

**Methods:**

370 preschoolers, aged 4–6 years, stratified according to gender, age and school region in the city of Londrina, PR, participated in the study. A questionnaire was applied to principals of preschools to analyze the school infrastructure and environment. Physical activity and sedentary behavior were estimated using accelerometers for five consecutive days during the children's stay at school. The odds ratio (OR) was estimated through binary logistic regression.

**Results:**

At school, regardless of age, preschoolers spend relatively more time in sedentary behaviors (89.6–90.9%), followed by light (4.6–7.6%), moderate (1.3–3.0%) and vigorous (0.5–2.3%) physical activity. The indoor recreation room (OR 0.20, 95%CI 0.05–0.83) and the playground (OR 0.08, 95%CI 0.00–0.80) protect four-year-old schoolchildren from highly sedentary behavior. An inverse association was found between the indoor recreation room and physical activity (OR 0.20, 95%CI 0.00–0.93) in five-year-old children. The indoor recreation room (OR 1.54, 95%CI 1.35–1.77), the playground (OR 2.82, 95%CI 1.14–6.96) and the recess (OR 1.54, 95%CI 1.35–1.77) are factors that increase the chance of six-year-old schoolchildren to be active.

**Conclusions:**

The school infrastructure and environment should be seen as strategies to promote physical activity and reduce sedentary behavior in preschool children.

## Resumo

**Objetivo:**

Analisar a atividade física e o comportamento sedentário de pré-escolares durante a permanência na escola e os fatores associados.

**Métodos:**

Participaram do estudo 370 pré-escolares de 4 a 6 anos, estratificados de acordo com sexo, idade e região da escola em Londrina (PR). Foi aplicado um questionário às diretoras das pré-escolas para analisar a infraestrutura e o ambiente escolar. A atividade física e o comportamento sedentário foram estimados com acelerômetros por cinco dias consecutivos durante a permanência na escola. A razão de chances (RC) foi estimada por meio da regressão logística binária.

**Resultados:**

Na escola, independentemente da idade, os pré-escolares permanecem relativamente mais tempo em comportamento sedentário (89,6%-90,9%), seguido de atividade física leve (4,6%-7,6%), moderada (1,3%-3%) e vigorosa (0,5%-2,3%). A sala de recreação interna (RC=0,20; IC95% 0,05-0,83) e o parque (RC=0,08; IC95% 0,00-0,80) protegem os alunos de 4 anos do comportamento sedentário elevado. Associação inversa foi encontrada entre sala de recreação interna e atividade física (RC=0,20; IC95% 0,00-0,93) nos escolares de 5 anos. Sala de recreação interna (RC=1,54; IC95% 1,35-1,77), parque (RC=2,82; IC95% 1,14-6,96) e recreio (RC=1,54; IC95% 1,35-1,77) são fatores que aumentam a chance dos escolares de 6 anos de serem ativos.

**Conclusões:**

A infraestrutura e o ambiente da escola devem ser considerados como estratégias para promover a atividade física e reduzir o comportamento sedentário em pré-escolares.

## Introduction

Sedentary lifestyle and physical activity are two health-related behaviors of preschool children. In children up to 4 years of age, sedentary behavior is an important factor for weight gain, increased LDL-cholesterol and decreased HDL-cholesterol levels.^1^ Similarly, there is a positive relationship between increased physical activity and increased bone density, better cardiometabolic profile and lower adiposity.^2^ It is recommended that children should spend a maximum of 2h per day being sedentary^3^ and achieve 180min/day of physical activity at any intensity.^4^

Children under 6 years of age attend preschools, with a daily average stay of 9h. For this reason, child care centers are no longer paternalistic and are committed to educating children, having as one of its actions the promotion of health^5^ through physical activity.^6^

Several studies describe the factors that increase the likelihood of preschoolers to engage in physical activity at school: playing in open spaces^7^ and playgrounds,^8^ engage in individual or in pairs activities,^8^ without the presence of adults,^7^ have toys and stuff to play with,^8^^,^ 9 promote opportunities for physical activity and instruct teachers in relation to physical activity.^9^ Environment Room with appliances such as TVs and video games,^9^ higher teacher-student relationship score, and no use of internal spaces for motor activities^10^ are associate with sedentary behavior.

In Brazil only one study investigated the association between the school environment and the weekly physical activity of preschoolers,^11^ and the only variable showing protection against low levels of physical activity was to have at least one recess time per day.^11^ There is no information related to sedentary behavior at school in Brazilian preschoolers. Furthermore, no study was conducted in Brazil assessing physical activity and sedentary behavior using accelerometry, an instrument that enables more precise measurement.

Due to the longer time that preschool children remain in school, lack of national studies, and inability to generalize the results of foreign studies, it is relevant to investigate the amount of physical activity and sedentary behavior during the school period and the environmental aspects in Brazilian preschoolers. Such information can guide intervention programs in order to increase physical activity, reduce sedentary behavior during their stay at school, and contribute to the fulfillment of the daily recommendations of these behaviors.

Thus, the aim of this study was to assess the amount of physical activity and sedentary behavior of preschoolers during their stay in the Municipal Centers of Early Childhood Education of Londrina (PR), as well as the associated factors.

## Method

Cross-sectional study performed in Municipal Centers of Early Childhood Education (Centros Municipais de Educação Infantil - CMEIs) of Londrina (PR). The Londrina municipal school system had 20 CMEIs with 1562 students enrolled in 2013, according to the City Department of Education. The CMEIs serve children up to 6 years of age, comprising nursery classes (Early Children Education 1: ECE1) pre-school (4-6 years old: ECE4 and ECE6). Children up to ECE5 study full-time and up to ECE6 only in a period of the day. The study sample was composed of students enrolled in ECE4, ECE5, and ECE6 classes.

Sample size calculation was made with the following parameters: students (*n*=1562), 5% sample error, 15% sample loss, 95% confidence interval, and 7% prevalence of moderate to vigorous physical activity at school.^12^ A design effect of 2 was applied due to the complex sample used, totaling 312 subjects. The sample size calculation was made with the Epi Info 7.0 software. Of the 581 children invited to participate in the study, 180 guardians refused to participate and 401 gave their written informed consent. Six children were absent in device placement day and 25 were excluded from analysis due to lack of valid data from accelerometers. The final sample consisted of 370 preschoolers.

The study sample was selected at random, with a cluster (school) stratified by sex, age, and city region. One school from each region was randomly selected and all students aged 4-6 years from the selected school were invited to participate. If the number of students in proportion to the region was not reached, another school in the area was selected. Eight schools were selected: one in the central region, two in the eastern region, one in the western region, three in the north region, and one in the southern region.

Inclusion criteria were: be enrolled and attending preschool; have no motor, physical, or mental difficulty that could prevent the accomplishment of the study procedures; and the informed consent form (ICF) signed by the legal guardian. The study was approved by the Institutional Review Board, Opinion No. 345,901, of the State University of Londrina, Londrina, PR, Brazil.

Before data collection, the study was approved by the Municipal Secretary of Education and by the school board. The first stage of data collection was an interview with the responsible for the school Board to evaluate the school environment. Information about the school environment were obtained through a questionnaire applied to the directors of the participating schools.^11^ The sections of the questionnaire were divided in order to gather information about physical education classes, recreational time, activities (sports tournaments, extracurricular physical activities, and sports), and the school physical facilities used for activities with preschoolers.

Anthropometric measurements of body weight and height for body mass index (BMI) calculation were collected on the day of accelerometer placement. A tape measure (Sanny, São Paulo, Brazil) and a digital scale with a precision of 100g (Plenna, MEA-03140, São Paulo, Brazil) were used. The standardization of measurements was performed according to previously described procedures,^13^ the cutoff points proposed by Conde and Monteiro were used.^14^ Parental education was estimated using the Abep questionnaire (2013),^15^ given with ICF to the children's parents or guardians.

To measure physical activity and sedentary behavior of preschoolers, we used ActiGraph GT3X accelerometers, dimensions 4.6×3.3×1.9cm, weight 19g, 16 MB memory, triaxial. Data collection took place in five consecutive days, physical activity was measured only during the period in which the children remained in school. Accelerometers were fixed at the waist, positioned on the left side by an elastic band. They were placed upon arrival of the child to school, with the start of data collection scheduled for 8h. It was taken at 5 pm each day, before the child's departure. For EC6 children, accelerometers were placed at 8 am and removed at 12 noon (morning class) or placed at 2 pm and removed at 6 pm (afternoon classes). All teachers responsible for CMEIs received previous explanations of the goals and methods of the study and training on the handling of the device.

To estimate physical activity and sedentary behavior, Epoch 1s was used to record the accelerometer information. The grouping of physical activity in light to vigorous (FALV) intensity was used due to the pattern of movement of preschoolers, who usually make quick, short movements, spending less time in vigorous physical activity and more time in sedentary behavior.^16^ In addition, the recommendation of physical activity for this age group is considered in any intensity.^4^ To meet the objectives of this study, the 75th percentile was adopted as cutoff points for physical activity and sedentary behavior.

The average use of accelerometers (min/day) considered valid was at least 360min for ECE4 and ECE5 children and 120min for ECE6 children. Children who had valid accelerometer data for at least three days were included in the analysis. To classify physical activity and sedentary behavior, the cutoff points of Sirard et al.^17^ were used for children 4 and 5 years old and the cutoffs proposed by Van Cauwenbrghe et al.^18^ for children aged 6 years.

All data collection was done by a single investigator. Data were described as mean, standard deviation, absolute and relative frequency. Chi-square test was used for bivariate analysis of the association between the school environment with the level of physical activity and sedentary behavior of preschoolers. Binary logistic regression analysis was used to assess odds ratio and 95% confidence intervals, gross and adjusted to sedentary behavior and physical activity. In multivariate analysis, the adjustment was made to environmental variables, sex and BMI of preschoolers. The variables with *p*≤0.05 in the multivariate analysis were considered significantly associated.

## Results

Of the 370 analyzed preschoolers, 50.4% were male, 2.8% were underweight, 72.7% were normal weight, 17% were overweight and 7.6% obese. Family socioeconomic status B2 (34.8%) and parents’ educational level less than eight years of schooling were the most frequent for all series (Table 1).

**Table 1:** Description of data on age, sex, nutritional status, parental education stratified by school grade, Londrina, PR, 2013.

	ECE4	ECE5	ECE6	All
	*n*=110	*n*=109	*n*=151	
*Age (years)*	3.7±0.4	5.2±0.3	6.1±0.3	5.2±0.8

*Sex (%)*				
Male	48.2	45.9	55.0	50.3

*Nutricional status (%)*				
Normal	75.4	80.8	72.2	75.6
Overweight	17.3	15.6	17.2	16.8
Obese	7.3	3.6	10.6	7.6

*Schooling ≥8 years (%)*				
Father	26.6	21.5	17.5	22.0
Mother	27.6	22.4	22.9	24.5

ECE, early childhood education.

The characteristics observed in the evaluated schools (*n*=8) are as follows: none of them had extracurricular activities, the same was seen regarding physical education classes; six schools offered recess time; one school offered two recess times per day, morning and afternoon; two schools offered four recess times, two in the morning and two in the evening; one school offered a recess time in the afternoon; and the other two schools reported no fixed schedules for recess. All schools that had recess allow children to play with toys for the activities. In two schools, the children shared space with children from other grades during recess, and only in one school this division took place during the entire recess period.

Of the analyzed schools, six had indoor recreation rooms, five had parks, and three had other facilities and portable toys used for physical activity. The reported indoor recreation rooms are “ludotecas” (educational, recreational, and cultural play areas), “brinquedotecas” (room of games and toys organized for the free use of children), video libraries, and libraries. The parks are places with fixed appliances for outdoor games, such as swings, slides, sandpit, marry-go-round, plus a small grassy area for leisure activities. One school in the central region reported having seesaws and jungle gym toys. Schools that reported having no park (*n*=3) had some portable equipment, such as slide, caterpillar tunnel, toy horses, giant snail toy, pool balls, tires, equipment for circuit and ropes. These schools had not park due to lack of physical space or because they were recently inaugurated.

All schools had covered patios, which were used for activities requiring more space in the schools. Older schools and close to the city center (*n*=3) had specific and broad area, while the newer schools or further away from the city center (*n*=5) used part of the covered dining room space for activities and, consequently, had a smaller space compared to other schools.

Regarding the results of physical activity and sedentary behavior in the school environment (Table 2), it is observed that, regardless of the grades, the students have the same level of physical activity, remain more time in sedentary behavior (89.6-90.9%), followed by light (4.6-7.6%), moderate (1.3-3.0%), and vigorous (0.5-2.3%) physical activity.

**Table 2:** Absolute and relative weekly participation in sedentary behavior and physical activity of different intensities in the school environment, stratified by school grade, Londrina, 2013.

	ECE4	ECE5	ECE6
	% - Mean±standard deviation in minutes		
Sedentary	89.6	90.9	90.1
	2234.5±352.7	2201.6±354.7	696.8±191.9
Light	7.6	7.0	4.6
	213.2±57.8	188.9±67.1	44.5±22.9
Moderate	2.3	1.3	3.0
	48.0±19.1	34.8±18.0	29.3±18.3
Vigorous	0.5	0.8	2.3
	13.9±9.5	15.3±10.0	20.8±17.4
LVFA	10.4	9.1	9.9
	275.2±78.0	238.9±89.9	94.6±56.0

LVFA, light to vigorous physical activity; ECE, early childhood education.

Significantly lower frequencies of sedentary behavior were found in ECE4 students attending schools that had indoor recreation room (53.6% vs. 81.7%) and park (25.0% vs. 74.4%). The associations remained after adjustment; the presence of indoor recreation area and park is a protective factor against high levels of sedentary behavior in ECE4 preschoolers. No associations were found between school environment and physical activity for children in ECE4.

There were no associations between school environment and sedentary behavior in ECE5 preschoolers. However, lower frequency of preschoolers above the 75th percentile for physical activity was found in children attending school with indoor recreation room (74.1% vs. 91.5%), the association remained in the multivariate analysis.

For ECE6 students, school environment was not associated with sedentary behavior in the adjusted analysis. On the other hand, it was found higher frequencies of students with physical activity above the 75th percentile in schools with indoor recreation room (100% vs. 64.6%), park (81.6% vs. 61.1%), and recess (100% vs. 64.6%). The results after adjusting for all variables in the model indicated that preschoolers who attend schools with indoor recreation room, park, and recess have a greater chance of being active than students who attend schools without these characteristics (Table 3).

**Table 3:** Association between infrastructure and school environment with sedentary behavior and physical activity of preschoolers stratified by school grade, Londrina, PR, 2013.

Variables	Preschoolers *n* (%)	Gross OR (95%CI)	*p*-value^a^	Adjusted OR (95%CI)	*p*-value
*EI4* - *Sedentary behavior*					
Indoor recreation room					
Yes	15 (53.6)	0.25 (0.10-0.65)	0.003	0.20 (0.05-0.83)	0.020
No	67 (81.7)	1.00		1.00	
Park					
Yes	7 (25.0)	0.11 (0.04-0.30)	0.000	0.08 (0.00-0.80)	0.014
No	61 (74.4)	1.00		1.00	

*ECE4* - *Physical activity*					
Indoor recreation room					
Yes	19 (70.4)	0.75 (0.28-1.98)	0.566	-	-
No	63 (75.9)	1.00		-	
Park					
Yes	18 (66.7)	1.32 (0.53-3.28)	0.551	-	-
No	50 (60.2)	1.00		-	

*ECE5 - Sedentary behavior*					
Indoor recreation room					
Yes	26 (96.3)	4.89 (0.61-39.34)	0.102	-	-
No	69 (84.1)	1.00		-	
Park					
Yes	15 (55.6)	0.64 (0.26-1.57)	0.336	-	-
No	54 (65.9)	1.00		-	

*ECE5 - Physical activity*					
Indoor recreation room					
Yes	20 (74.1)	0.26 (0.08-0.84)	0.019	0.20 (0.04-0.93)	0.030
No	75 (91.5)	1.00		1.00	
Park					
Yes	17 (63.0)	0.98 (0.39-2.41)	0.966	-	-
No	52 (63.4)	1.00		-	

*ECE6 - Sedentary behavior*					
Indoor recreation room					
Yes	31 (81.6)	1.82 (0.73-4.56)	0.193	-	-
No	80 (70.8)	1.00		-	
Park					
Yes	32 (84.2)	0.28 (0.11-0.73)	0.007	-	-
No	68 (60.2)	1.00		-	
Recess					
Yes	31 (81.6)	1.82 (0.73-4.56)	0.193	-	-
No	80 (70.8)	1.00		-	

*ECE6 - Physical activity*					
Indoor recreation room					
Yes	38 (100.0)	1.54 (1.35-1.77)	0.000	1.58 (1.29-1.93)	0.015
No	73 (64.6)	1.00		1.00	
Park					
Yes	31 (81.6)	2.82 (1.14-6.96)	0.021	1.45 (1.16-1.82)	0.011
No	69 (61.1)	1.00		1.00	
Recess					
Yes	38 (100.0)	1.54 (1.35-1.77)	0.000	1.58 (1.29-1.93)	0.015
No	73 (64.6)	1.00		1.00	

a *p*, chi-square test.

ECE, early childhood education; OR, odds ratio; 95%CI, 95% confidence interval; OR adjusted for the environmental, infrastructure, gender, and BMI variables.

## Discussion

To the best of our knowledge, this is the first study to assess physical activity and sedentary behavior of Brazilian preschoolers during their stay in school using accelerometry. The main results indicated that physical activity at school offers a poor contribution to the amount of daily physical activity recommended for children. In addition, schools with indoor recreation room and park protect younger preschoolers against sedentary behavior. Older preschoolers who attend schools with indoor recreation room, park, and recess time are more likely to be active compared to those attending schools without such infrastructure.

The levels of physical activity and sedentary behavior seen in preschools are supported by previous reports. They indicate that children spend most of the time at school in sedentary behavior (50-94%), followed by light physical activity (5-27%) and moderate to vigorous activity (1-17%).^7^^,^ 8 ^,^ 10 ^,^ 12 ^,^ 19 These results may be explained by the fact that for this age group there are loads of indoor activities in schools under supervision, in order to stimulate diverse cognitive and motor learning, particularly basic literacy, which result in high permanence in sedentary behavior. Although sedentary behavior is predominant during their stay in schools due to the characteristics of the activities, the results of our study are alarming, since the values are among the highest in previous studies.^7^^,^ 8 ^,^ 10 ^,^ 12 ^,^ 19

One aspect to be considered is the contribution of the physical activity done at school to attend the weekly children's physical activity recommendation. This recommendation suggests 180min of daily physical activity at any intensity.^4^ Considering the working days, as children attend school only in those days, a child should accumulate 900min of physical activity per week. In the present study, ECE4, ECE5, and ECE6 accumulated on average 275.2, 238.9, and 94.6min per week, which is a contribution of 30.6%, 26.5%, and 10.5%, respectively, for the recommendation. For children in ECE4 and ECE5, the results are worrying because the child remains in school in the morning and afternoon periods, and probably will not do the amount of physical activity that remains to achieve the recommendation at night. As a result, there is a high prevalence of preschoolers with low levels of physical activity (60%),^20^ which exposes the children to different health risks.^1^^,^ 2

In addition to the recommendation of daily physical activity, there is recommendation for physical activity at school. Preschool child should engage in at least 60min of structured physical activity, 60min of unstructured activity, and less than 60min of sedentary behavior, except when sleeping.^6^ The preschoolers analyzed do not meet this recommendation, since the children in ECE4, ECE5, and ECE6 accumulate, respectively, 275.2, 238.9, and 94.6min of weekly physical activity rather than at least 600min. These results demonstrate that, as reported in another study,^21^ preschools have contributed little to promote physical activity and decrease sedentary behavior.

The association between the school environment with physical activity and sedentary behavior has been assessed in preschoolers.^9^^,^ 10 ^,^ 22 The results showed that having an indoor recreation room and park protects children in ECE4 against sedentary behavior because it allows consistent motor activities, such as games and activities that encourage preschoolers not to remain still.^10^ However, despite protecting younger preschoolers against sedentary behavior, the activities do not provide enough movement to be classified as physical activity.

Our results for sedentary behavior in ECE4 support those of studies performed in Australia,^10^ Canada,^22^ and United States.^9^ The aspects that protect children's sedentary behavior are promoting opportunities for physical activity, training for teachers and staff regarding physical activity, use of indoor space for motor activities, and have additional fixed equipments,^9^^,^ 10 while the stimulus to sit, watch TV, or play video games increases the sedentary behavior of American preschoolers.^9^^,^ 22

As for physical activity in preschoolers in ECE6, the results showed that having indoor recreation room, park, and recess promotes light to vigorous physical activity. Studies performed in other countries have shown that many aspects of a school, such as having mobile devices, opportunities for students to do physical activity, open space, balls and mobile toys, lawns, parks, and use of indoor spaces for motor activity are positively associated with preschoolers physical activity.^7^^-^ 10 ^,^ 23 Two aspects may explain the association between school environment and physical activity only in the older preschoolers. At the age of six years, children reach the mature stage of the fundamental motor skills,^24^ as well as perform better in fine motor skills, overall motor skills, balance, body structure, and spatial organization,^25^ which gives them greater autonomy and hence more intensity in their activities. This could be seen in this study, since, although ECE6 preschoolers remained only a period of the day at school, they are on average engaged in a greater amount of vigorous activity (20.8min) compared to ECE4 (15.3min) and ECE5 (13.9min). Moreover, moderate and vigorous activity in ECE6 is more intense (2.3%) than in ECE4 (0.5%) and ECE5 (0.8%). Another factor that may explain the association between physical activity and environment only in ECE6 is children monitoring. The activities structured by adults result in less physical activity of moderate to vigorous intensity compared to those structured by children.^7^^,^ 26 Similarly, supervision can inhibit the physical activity of preschoolers.^26^ In our study, supervision of students has not been evaluated. However, probably due to the greater autonomy of the older preschoolers, it is possible that there was less supervision of children and, consequently, greater use of the school environment for physical activity.

The present study advances knowledge on physical activity and sedentary behavior, as well as on associated environmental factors in preschoolers. All investigations related to this theme were made outside Brazil,^7^^-^ 12 ^,^ 16 ^,^ 19 ^,^ 22 ^,^ 23 ^,^ 26 and socio-cultural and infrastructure differences make it difficult to generalize the results. The use of accelerometers allowed the identification of both sedentary behavior and physical activity at different intensities in a representative sample and increased the external results validity. On the other hand, the absence of direct observation prevented estimating physical activity and sedentary behavior in each activity and school environment. Additionally, the present study did not investigate the dimensions of each school environment—a factor that prevented the assessment of the association between the size of the areas, physical activity, and sedentary behavior.

According to the results of this study, it is concluded that in approximately 10% of the time at school children are engaged in physical activities and in the remaining time they are sedentary. Indoor recreation room and park protect young preschoolers against high sedentary behavior, while the presence of indoor recreation room, park, and recess increase the chance of older schoolers being active.
